# Biological impact of recurrent sexually transmitted infections on HIV seroconversion among women in South Africa: results from frailty models

**DOI:** 10.7448/IAS.18.1.19866

**Published:** 2015-04-24

**Authors:** Handan Wand, Gita Ramjee

**Affiliations:** 1 The Kirby Institute, University of New South Wales, Kensington, Australia; 2 HIV Prevention Unit, Medical Research Council, Durban, South Africa

**Keywords:** South Africa, HIV incidence, recurrent sexually transmitted infections, joint modelling

## Abstract

**Introduction:**

Understanding the impact of curable sexually transmitted infections (STIs) on HIV transmissibility is essential for effective HIV prevention programs. Investigating the impact of longitudinally measured recurrent STIs on HIV seroconversion is the interest of the current paper.

**Methods:**

In this prospective study, data from a total of 1456 HIV-negative women who enrolled in a HIV biomedical trial were used. It was hypothesized that women who had recurrent STI diagnoses during the study share a common biological heterogeneity which cannot be quantified. To incorporate this “unobserved” correlation in the analysis, times to HIV seroconversion were jointly modelled with repeated STI diagnoses using Cox regression with random effects.

**Results and discussion:**

A total of 110 HIV seroconversions were observed (incidence rate of 6.00 per 100 person-years). In a multivariable model, women who were diagnosed at least once were more likely to seroconvert compared to those who had no STI diagnosis [hazard ratio (HR): 1.63, 95% confidence interval (CI): 1.04, 2.57]; women who had recurrent STI diagnoses during the study were 2.5 times more likely to be at increased risk of HIV infection (95% CI: 1.35, 4.01) with an estimated *frailty* variance of 1.52, with *p<*0.001, indicating strong evidence that there is a significant correlation (heterogeneity) among women who had recurrent STIs. In addition to this, factors associated with incidence of STIs, namely not being married and having a new sexual partner during the study follow-up, were all significantly associated with increased risk for HIV seroconversion (HR: 2.92, 95% CI: 1.76, 5.01 and HR: 2.25, 95% CI: 1.63, 3.83 respectively).

**Conclusions:**

The results indicated that women who were at risk for STIs were also at risk of HIV infection. In fact, they share the similar risk factors. In addition to this, repeated STI diagnoses also increased women’s susceptibility for HIV infection significantly. Decreasing STIs by increasing uptake of testing and treatment and reducing partner change plays a significant role in the trajectory of the epidemic.

## Introduction

It is widely accepted that a woman’s risk of HIV infection is increased by diagnosis with sexually transmitted infections (STIs). Chlamydia, gonorrhoea and syphilis have been linked consistently to increased HIV acquisition [1–4]. In addition to the strong biological associations between these pathogeneses, they also share common socio-demographic and sexual risk behaviours [5,6]. For this reason, STI prevention is often included as part of HIV prevention packages [7].

The current study was motivated by a HIV prevention trial which was designed to investigate the effect of a microbicide on a cohort of women in South Africa [7]. Socio-economic characteristics and sexual behaviours of the women were collected. They were also tested for HIV and other STIs at pre-scheduled clinic visits. Time to progression to STI(s) and HIV infection were also recorded.

Standard statistical techniques such as Cox regression are commonly used to analyze time to event data but they do not account for potential correlations among individuals who experience similar events [8]. For example, our study population got tested for STIs (*recurrent events*) and HIV (*terminating event*) at every study visit during the study follow-up. It was hypothesized that recurrent STIs may increase women’s susceptibility for HIV acquisition. In this setting, we assumed that individuals with one or more STI diagnoses during the study may be correlated because of some “unmeasured” biological changes due to these *recurrent events*; representing the accumulation of effects on hazard of transition which have not been measured or are difficult to quantify. Intuitively, we assumed that women who had recurrent STIs during the study have similar life style and/or biological characteristics.

To the best of our knowledge, this is the first comprehensive study to examine the impact of repeated STI(s) on HIV seroconversion among a large group of women who are known to be at high risk of HIV infection.

## Methods

A detailed description of the study population was described elsewhere [7]. Briefly, Carraguard^®^ was a randomized, placebo-controlled, double-blind trial, which was designed to investigate the efficacy and long-term safety of the candidate, that is, a carrageenan-based compound for the prevention of HIV infection among sexually-active, HIV-negative women, aged 16 years or older, in African sites. The trial participants, who were randomly assigned by a block randomization scheme to Carraguardor placebo, were instructed to use one applicator of gel plus a condom during each vaginal sex act. Participants were followed up for up to two years. Visits every three months included testing for HIV presence and pregnancy, pelvic examinations, risk reduction counselling and treatment for curable STIs and symptomatic vaginal infections. The primary outcome was time to HIV seroconversion [7]. Time to event was measured based on a discrete time scale determined by an individual’s quarterly visit. For women who tested positive, the time of infection was defined as the time of first positive STI test result. For cases in which one or more visits were missed in the intervals between the last negative and first positive tests, the time of infection was assumed to be the visit containing the midpoint between these two time points.

Rapid HIV-1 blood testing was used during the study follow-ups. A woman’s HIV serostatus was confirmed with parallel HIV-1 rapid tests, and positive/discordant tests were confirmed by third-generation enzyme immunoassay or polymerase chain reaction for the detection of HIV-1 ribonucleic acid. “Women were also tested for curable STIs—including chlamydia, gonorrhoea, syphilis, and trichomoniasis, those who tested positive were treated as part of the study protocol” [7]. Repeated STIs were defined as a positive test result for any of the curable STIs (chlamydia, gonorrhoea, syphilis and trichomoniasis) occurring in next testing (i.e. three months) after an initial positive result.

Age at baseline (in quartiles), marital status, new sexual partner(s) during the study (yes/no), average weekly number of sexual acts (three or more versus less than three), diagnosis of a sexually transmitted infection (at baseline), unprotected oral/anal sex in past three months, using any form(s) of contraceptive were all used as binary variables. Since more than 99% of the women reported having a regular partner, this information was not among the risk factors considered in the analysis.

## Statistical analysis

In this context, repeated STIs were considered as *recurrent events* and HIV seroconversion was assumed to be the *terminating event*. Participants’ observed characteristics such as socio-demographic and sexual behaviours were incorporated into the models used in this study. In addition to this, a random person component, *frailty*, represents the accumulation of effects on hazard of *recurrent STIs* which have not been measured or are difficult to quantify. It was hypothesized that recurrent STIs may increase women’s susceptibility for HIV acquisitions. In this setting, we assumed that individuals with one or more STI diagnoses during the study may be correlated because of some “unmeasured” biological changes due to these recurrent events, representing the accumulation of effects on hazard of transition which have not been measured or are difficult to quantify. Intuitively, we assumed that women who had recurrent STIs during the study will have similar biological characteristics.

In Model 1 (see below), the outcome variable was the time to the first STI diagnosis. Standard univariable and multivariable Cox regression models were used to determine the significant predictors of incidence of STI in Model 1, whereas times to HIV incidence were jointly modelled with times to STI(s) diagnosis (Model 2, see below). The formulation of Model 2 allows us to assess subject heterogeneity due to the repeated STIs. In this setting, unobserved heterogeneity (i.e. frailty) was incorporated into the model as random component acting multiplicatively on the baseline hazard function and was assumed to follow a Gamma distribution. The outcome/censoring indicators were *D*_1*j*_=1 for STI(s) and *D*_2*j*_=1 for HIV seroconversion (zero otherwise). If HIV seroconversion occurs without any STI diagnoses, then the time to HIV seroconversion (or censoring) is taken to be the time to HIV seroconversion but *D*_1*j*_=0 corresponding to a censored observation.

**Model 1:**
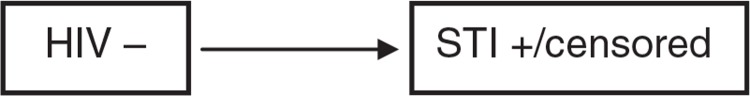
Time to STI (first) diagnosis (before HIV diagnosis)

**Model 2:**
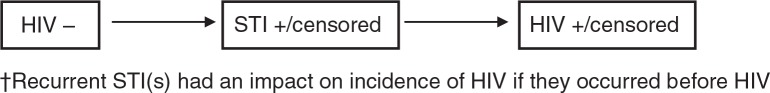
Time to HIV seroconversion before/after STI(s) diagnosis^†^

The proportionality of the hazard ratios (HRs) was assessed using the Stata 12.0 function called “estat phtest” (for individual covariates and globally) [9]. The proportionality assumption was not violated in any of the models considered (global *p*-values were estimated to be 0.0616 and 0.6827 for the STI and HIV acquisition models, respectively). Covariates were entered into the multivariate model if they had a *p*-value of less than 0.10 in the univariate analysis. The final multivariate model was determined using a forward step-wise approach and included statistically significant covariates (*p<*0.05).

## Results and discussion

A total of 1456 HIV uninfected women consented to be enrolled in the study. During the study follow-up, 110 HIV seroconversions were observed with the crude incidence rate of 6.00 per 100 person-years [95% confidence interval (CI): 5.0, 7.2] (data not shown). The two models, designated Model 1 (for time to STI), and Model 2 (for time to HIV infection) as described in the previous section were fitted and the results were reported in Tables 1 and 2, respectively. In multivariable analysis, diagnosis with STI(s) at baseline, younger age (<23 years old, first quartile cut-point), not being married and reporting a new sexual partner during the study were all determined to be significantly associated with increased risk for incidence of STIs (HR: 2.34, 95% CI: 1.85, 2.96, HR: 1.79, 95% CI: 1.29, 2.49, HR: 1.50, 95% CI: 1.10, 2.06 and HR: 1.47, 95%: CI: 1.08, 2.00) (Table 1).

**Table 1:** Univariable and multivariable survival models for STI incidence: results from Model 1

	Risk factors for STI incidence				
	
		Univariable analysis		Multivariable analysis	
	
	Case/*n*	HR (95% CI)	*P*	HR (95% CI)	*P*
STI at screening					
No	214/1151	1		1	
Yes	109/305	2.53 (2.01, 3.19)	*<*0.001	2.34 (1.85, 2.96)	<0.001
Age groups					
< 23 (1st quartile)	117/432	2.28 (1.70, 3.07)	*<*0.001	1.79 (1.29, 2.49)	<0.001
23–27 (2nd quartile)	57/308	1.33 (0.94, 1.88)	0.107	1.14 (0.79, 1.64)	0.488
28–37 (3rd quartile)	74/384	1.21 (0.88, 1.69)	0.238	1.15 (0.83, 1.60)	0.407
38+ (4th quartile)	75/332	1		1	
Marital status					
Not married	61/382	2.10 (1.59, 2.79)	*<*0.001	1.50 (1.10, 2.06)	0.012
Married	262/1074	1		1	
New partner during the study					
No	274/1307	1		1	
Yes	49/149	1.76 (1.30, 2.19)	*<*0.001	1.47 (1.08, 2.00)	0.015
Condom used during the last sexual act					
No	171/1076	1			
Yes	66/380	1.30 (0.97, 1.71)	0.100	–	
Using any forms of contraceptive					
No	82/444	1			
Yes	244/1012	1.16 (0.90, 1.49)	0.246	–	
Unprotected oral sex in past three months					
No	266/1238	1			
Yes	57/218	1.40 (1.05, 1.86)	0.021	–	
Unprotected anal sex in past three months					
No	306/1376	1		–	
Yes	17/80	1.07 (0.65)	0.797	–	
Average number of sexual acts					
< 3	209/917	1			
3 or more	114/539	0.90 (0.72, 1.13)	0.381	–	

**Table 2:** Univariable and multivariable survival models for HIV incidence: results from Model 2

	Risk factors for HIV incidence				
	
		Univariable analysis		Multivariable analysis	
	
	Case/*n*	HR (95% CI)	*P*	HR (95% CI)	*P*
STI at screening					
No	83/1151	1		–	
Yes	27/305	1.24 (0.80, 1.92)	0.327	–	
STI recurrent					
No infection at any point	38/799	1		1	
One-infection	37/414	1.78 (1.13, 2.79)	0.013	1.63 (1.04, 2.57)	0.034
Recurrent infection^a^ a	21/151	2.64 (1.55, 4.50)	*<*0.001	2.51 (1.35, 4.01)	<0.001
Age (in quartiles)					
< 23 (1st quartile)	48/432	3.74 (2.10, 6.64)	*<*0.001	3.49 (2.05, 5.96)	<0.001
23–27 (2nd quartile)	29/308	3.00 (1.62, 5.56)	*<*0.001	2.75 (1.55, 4.87)	0.001
28–37 (3rd quartile)	17/384	1.27 (0.64, 2.52)	0.496	1.23 (0.67, 2.24)	0.503
38+ (4th quartile)	16/332	1		1	
Marital status					
Not married	14/382	2.96 (1.69, 5.19)	*<*0.001	2.92 (1.76, 5.01)	<0.001
Married	96/1074	1		1	
New partner during the study					
No	84/1307	1		1	
Yes	26/149	2.72 (1.75, 4.22)	*<*0.001	2.25 (1.63, 3.83)	<0.001
Condom use during the last sexual act					
No	77/1076	1			
Yes	33/380	1.35 (0.89, 2.02)	1.54	–	
Using any form of contraceptive					
No	31/444	1			
Yes	79/1012	1.07 (0.71, 1.62)	0.744	–	
Unprotected oral sex in past three months					
No	89/1238	1			
Yes	21/218	1.45 (0.90, 2.34)	0.123	–	
Unprotected anal sex in past three months					
No	102/1376	1			
Yes	8/80	1.51 (0.74, 3.11)	0.260		
Average weekly number of sexual acts					
< 3	72/917	1		–	
3 or more	38/539	1.11 (0.76, 1.61)	0.584		
Variance of frailty	–	–		1.52 (1.40, 1.65)	<0.001

a One positive test with at least one positive result previously.

Table 2 presented the results from the fitting the random effects model by allowing a random component (i.e. frailty) in the model. In the multivariable model, compared to those without any STI diagnosis, women who were diagnosed once were more likely to seroconvert compared to those who had no STI diagnosis (HR: 1.63, 95% CI: 1.04, 2.57). Women who had recurrent STI diagnoses during the study were 2.5 times more likely to be at increased risk for HIV infection (HR: 2.51, 95% CI: 1.35, 4.01) compared with the estimated *frailty* variance of 1.52 (95% CI: 1.40, 1.65), with *p<*0.001, giving evidence that there is a significant correlation (heterogeneity) among women who had recurrent STI. Consistent with the results from Model 1, other factors such as not being married and having a new sexual partner during the study follow-up were all significantly associated with increased risk for HIV seroconversion (HR: 2.92, 95% CI: 1.76, 5.01 and HR: 2.25, 95% CI: 1.63, 3.83). In this study, the frailty component was assumed to have a Gamma distribution because of the mathematically attractive properties. The analysis was repeated using the log-Normal distribution, but the results were almost the same.

Sexual and reproductive tract infections have been consistently shown to be associated with HIV acquisition [1–4]. Transmission of HIV is presumed to be facilitated by these infections through disruption or inflammation of the genital epithelium [5,6]. In addition to this biological plausibility, they share similar risk behaviours such as unprotected sex. Therefore, measurements on individuals who experience these recurrent events may be correlated. Standard survival models such as Cox regression models, based on homogeneity of the study population, may not be suitable as they may produce biased (incorrect) estimates. This is particularly problematic in clinical trials as they are generally designed assuming homogeneity among study participants. Modelling of the potentially correlated events should account for potential correlations between them. Frailty models are a natural way of analyzing the correlated survival data by accounting for this unmeasured heterogeneity.

In this study, we jointly modelled the *recurrent events* (i.e. STIs) and a *terminating event* (i.e. HIV infection) using random effect models in survival analysis setting [10,11]. The models included a random component (i.e. frailty) which disentangled the accumulation effect of an unobserved biological factor due to these recurrent events. Results from our study indicated that younger age, not being married and changing partner during the study were all significantly associated with increased risk for STI(s). Impact of recurrent STIs was assessed after adjusting for unobserved biological heterogeneity of those who had repeated STIs during the study. Consistent with what is known about these two study outcomes, the same variables were associated with increased risk for STIs and HIV infection. In addition to this, after adjusting for biological risk factors (i.e. heterogeneity), women who had recurrent diagnoses with STIs were determined to be 2.5 times likely to seroconvert compared to those who did not have any diagnoses; when the heterogeneity was ignored, impact of recurrent STIs was slightly underestimated (HR: 1.97 versus HR: 2.51). However, overlapping CIs broadly indicated that this difference may not be statistically significant.

The standard Cox regression model does not contain a mechanism that can cope with potential correlation between the failure times within the same subject. This type of model and its extensions have been widely used to model correlated events [12,13]. Our study was motivated by the undisputable complex and multifactorial associations between HIV infection and other curable STIs [14–17]. Biological and behavioural factors have been consistently reported to be sources of these associations and ignoring them may mask their real effect and potentially undermine the validity of the study findings.

Condom use was not determined to be a risk factor for STIs and HIV infections. However, it is widely accepted that STIs and HIV acquisition can only occur during unprotected sex, identifying recurrent STIs as a significant risk factor for HIV infection give a strong evidence for low-level condom use in this population (likely much lower than it was reported). In addition to this, not being married and having a new partner during the study follow-up are also indications for multiple/concurrent sexual partners. There is evidence that sexual behaviours developed in adolescence continue later in life [17–19]. Therefore, public health interventions are required to target women at early ages to reduce high-risk sexual behaviours before they reach their sexual peak.

Our results should be interpreted under several limitations. Our study population was the group of women who enrolled in a HIV prevention trial. Therefore, results from the study may not be generalizable. In addition to this, the majority of the demographic and sexual behaviours were self-reported. Therefore, they may be subject to recall bias and/or misclassification. We would also like to note that effects of other unmeasured characteristics, including poverty, concurrent sex partners and commercial sex work, may have an impact on the results. No data concerning migration and sexual behaviour of male partners were available or included in this study.

## Conclusions

The current study indicated that women who were at risk for STIs were also at risk for HIV. However, recurrent STI diagnoses may greatly increase women’s susceptibility for HIV infection [20–23]. Therefore, along with other high-risk sexual behaviours, preventing and treating STIs may reduce the number of HIV acquisitions based on various reasons.

## Fundings

The US Agency for International Development (HRN-A-00–99–00010 and GPO-A-00–04–00019) and the Bill & Melinda Gates Foundation (grant number 18057) funded the Carraguard^®^ study.
